# Collagen Extract Derived from Yeonsan Ogye Chicken Increases Bone Microarchitecture by Suppressing the RANKL/OPG Ratio via the JNK Signaling Pathway

**DOI:** 10.3390/nu12071967

**Published:** 2020-07-02

**Authors:** Kaudjhis Patrick Ulrich N’deh, Han-Seok Yoo, Kang-Hyun Chung, Kwon-Jai Lee, Dong-Hee Kim, Jin A Yoon, Jeung Hee An

**Affiliations:** 1 Department of Food Science and Technology, Seoul National University of Science & Technology, Seoul 01811, Korea; kaudjhispatrick@gmail.com (K.P.U.N.); 0223yhs@naver.com (H.-S.Y.); carl@seoultech.ac.kr (K.-H.C.); 2 Department of Food Science and Nutrition, KC University, Seoul 07661, Korea; yoonjina@hanmail.net; 3 Department of Advanced Materials Engineering, Daejeon University, Daejeon 34520, Korea; jmul@ssu.ac.kr; 4 Department of Oriental Medicine, Daejeon University, Daejeon 34520, Korea; dhkim@dju.ac.kr

**Keywords:** *Gallus gallus domesticus*, osteoblastogenesis, osteoclastogenesis, osteoporosis, ovariectomized rat

## Abstract

Yeonsan Ogye is a traditional Korean chicken breed *(Gallus domesticus*, GD), with a dominant gene for fibromelanosis, showing entirely black fluffy head feathers, ear lobes, and pupils. GD collagen extract (78.6 g per 100 g total protein) was derived from the flesh of Yeonsan Ogye. The effects of GD collagen on bone mass, microarchitecture, osteogenic, osteoclastogenic differentiations, and function factor expression were investigated in ovariectomized (OVX) rats. GD collagen stimulated osteogenesis in OVX rats and increased tibial bone strength and calcium content. Micro-computed tomography analysis of tibia cross-sections revealed that GD collagen attenuated the OVX-induced changes in trabecular thickness, spacing, and number. GD collagen stimulated alkaline phosphatase activity, bone-specific matrix proteins (alkaline phosphatase (ALP), osteocalcin, collagen type I (COL-I)) and mineralization by activating bone morphogenetic protein 2 (BMP-2)/mothers against decapentaplegic homolog 5 (SMAD5)/runt-related transcription factor 2 (Runx2). GD collagen inhibited osteoclast differentiation and function gene markers (TRAP, cathepsin K) by interfering with the Wnt signaling, increasing OPG production, and reducing the expression of RANKL, TRAP, and cathepsin K. GD collagen promoted osteogenesis by activating the p38 signal pathway and prevented osteoclastogenesis by lowering the RANKL/OPG ratio and blocking the JNK signaling pathway. Dietary supplementation with GD collagen might inhibit osteoclastogenesis, stimulate osteoblastogenesis, and regulate bone metabolism.

## 1. Introduction

Bone is a highly mineralized connective tissue that continually undergoes resorption of the bone matrix by osteoclasts and deposition of the osteoid matrix (containing ~90% of collagen type I) by osteoblasts to maintain the skeleton’s structural integrity [1,2]. Imbalance triggered by increasing bone matrix resorption, without corresponding new bone tissue formation, can lead to bone loss, quality deterioration, microarchitecture, and osteoporosis [3]. Traditional first-line prevention and treatment of osteoporotic fractures, such as hormone replacement therapy, have several side effects in chronic use [4,5]. Therapeutic agents in osteoporosis, such as bisphosphonates and modulators of estrogen and estrogen receptors, delay bone loss by inhibiting osteoclast activity or differentiation; however, they showed limited efficacy in promoting new bone formation [6,7]. Interest is growing in alternative therapeutics for the prevention and treatment of osteoporosis. Preferred for having fewer side effects, natural products include traditional Chinese medicine, herbal treatments, vitamins D and C, and collagen extract. They promote bone formation by activating osteoblastic proliferation and differentiation, by expressing bone-specific matrix proteins (alkaline phosphatase (ALP), bone sialoprotein (BSP), osteocalcin (OCN), osteopontin (OPN), and collagen type I (COL-I)), transcription factor (runt-related transcription factor 2 (Runx2), core-binding factor α1 (Cbf α1), and SMAD 1/5/8, osterix(Osx)), signal pathways (MAPK, bone morphogenetic protein (BMP)), OPG/RANKL system of osteoblasts, and estrogen-like biological activities [8,9,10]. Along with the activation of osteoblastogenesis, some natural products also suppress osteoclastogenesis by inhibiting the expression of the receptor activator for cathepsin K, receptor activator of nuclear factor kappa-B ligand (RANK-L), tartrate-resistant acid phosphatase (TRAP), and the regulator of osteoclast differentiation, such as proto-oncogene c-Fos and Nfatc1 [11,12].

Over the past few decades, animal sources of collagen, from fish, pigs, cows, and chickens, have been used to alleviate osteoarthritis and osteoporosis symptoms [13,14,15,16,17]. Collagen-derived peptides are thought to interact with the bone matrix to stimulate bone anabolism [17]. Collagen supplements enhance bone mineral density (BMD), exhibit osteogenic activity by promoting osteoblast differentiation, activating osteoblast-mediated bone formation, and protecting articular and cartilage health [18,19]. The primary culture of murine bone cells exposed to bovine, porcine, or fish collagen hydrolysate (2 kDa; 0.2. 1.0 mg/mL) exhibited a dose-dependent increase in ALP [17]. Oral administration of shark skin collagen extract has increased the BMD of the femur epiphysis, as well as enhanced the quantity of COL-1 and proteoglycans in the epiphysis of ovariectomized (OVX) rats [14]. Collagen supplements in the diets of female OVX rats (0.416 g of CH/day; 8 weeks feed) induced higher levels of protein content and OCN [18]. Oral ingestion of collagen hydrolysates with calcitonin substantially inhibited bone resorption in patients with postmenopausal osteoporosis, compared to calcitonin administration alone [15]. However, only a few studies have shown the collagen extract-induced stimulation of new bone tissue via the inhibition of osteoclastic differentiation and osteoclast-driven bone resorption.

This study aimed to investigate bone metabolism regulation in vitro and in vivo, using GD collagen. This collagen extract was derived from the flesh of Yeonsan Ogye, the entirely black Korean chicken breed *Gallus gallus domesticus.* We evaluated the proximate chemical content of the GD collagen and deducted the collagen content from the total protein. Next, we assessed the in vitro osteogenesis and anti-osteoclastogenic effects of GD collagen, using a human MG-63 osteoblast-like cell and osteoclast precursor RAW 264.7 cells. We also created a rapid animal model of osteoporosis by combining ovariectomy (OVX) with a low calcium diet in rats to study the in vivo effects of two dosages of GD collagen, GDC2 and GDC3. Finally, we investigated the potential mechanisms of GD collagen in the OVX rat model.

## 2. Materials and Methods

### 2.1. Preparation and Composition of GD Collagen

Korean black GD chicken, called Yeonsan Ogye, was obtained from Jisan Plantation (Chungnam, Korea) and chopped. GD collagen was extracted from the flesh with water in a pressure chamber at 121 °C and 0.5 MPa for 30 min. The extract was filtered using Whatman No. 2 filter paper, and a vacuum evaporator was used for the filtrate concentration. Concentrated extracts were lyophilized by freeze-drying (IL Shin, Seoul, Korea) and stored at −80 °C until use.

The proximate chemical composition of GD collagen, including total protein, lipid, ash, and moisture contents (g/100 g of GD collagen) were evaluated and reported as percentage composition, using the standard methods of the Association of Official Analytical Chemists (AOAC) [20]. Total carbohydrate content was estimated by the difference [100 − (moisture + protein + fat + ash)]. The total collagen content (g/100 g total protein) was evaluated by the hydroxyproline assay, as described in previous studies [21,22].

### 2.2. Cell Culture

MG-63 osteoblast-like cells and osteoclast precursor RAW 264.7 cells (Korean Cell Bank, Seoul, Korea) were cultured in Roswell Park Memorial Institute (RPMI) medium or Dulbecco’s modified Eagle’s medium (DMEM), containing 10% fetal bovine serum (Hyclone, Logan, UT, USA) and 1% penicillin-streptomycin (GIBCO, Grand Island, NY, USA), in a 5% CO_2_ incubator at 37 °C.

### 2.3. Cell Vviability Assay

MG-63 cells and RAW 264.7 cells (5 × 10^4^) were seeded in a 96-well plate and incubated for 24 h. Cell viability was determined using the 3-(4,5-dimethylthiazol-2-yl)-2,5-diphenyltetrazolium bromide (MTT; Promega, Madison, WI, USA) assay, as described in previous studies [23].

### 2.4. Analysis of ALP Activity

ALP activity was measured as the rate of p-nitrophenyl phosphate (pNPP) hydrolysis. MG-63 cells (5 × 10^4^) were seeded in 96-well plates, incubated in RPMI medium for 24 h, and then treated with the indicated concentrations of GD collagen for 96 h. The cells were lysed in 0.1% Triton X-100 and incubated with pNPP at 37 °C for 60 min. ALP activity was determined based on the optical density measured at 405 nm, using an Asys UVM 340 microplate reader (Biochrom Ltd., Cambridge, UK). 

### 2.5. Analysis of Mineralization by Adherent Cells in Culture Using Alizarin Red S Staining

The degree of mineralization was analyzed after a 14-day treatment with GD collagen. The MG-63 cells were washed with phosphate-buffered saline (PBS) and fixed with 70% ethanol for 1 h, prior to staining with 40 mM alizarin red S in deionized water (pH 4.2) for 15 min. After aspiration of the alizarin red S solution, the cells were incubated in PBS for 15 min in an orbital rotator, rinsed once with fresh PBS, and de-stained for 15 min with 10% (w/v) cetylpyridinium chloride in 10 mM sodium phosphate (pH 7). Finally, the extracted stain was transferred to a 96-well plate, and the absorbance values were measured at 550 nm, using an Asys UVM 340 microplate reader.

### 2.6. TRAP Activity and Staining

RAW 264.7 cells (5 × 10^4^) were seeded in 96-well plates containing DMEM with 10% fetal bovine serum. Four days after stimulation of the cells with 50 ng/mL RANKL, the cells were washed with PBS. To measure the TRAP activity, the cells were fixed in 3.5% formaldehyde for 10 min, washed with distilled water, and incubated in 50 mM citrate buffer (pH 4.5) containing 10 mM sodium tartrate and 6 mM pNPP. After a 1-h incubation, the mixtures were moved into new 96-well plates containing an equal volume of 0.1 N NaOH, and the absorbance values were measured at 405 nm using an Asys UVM 340 microplate reader. For TRAP staining, the cells were stained using a leukocyte acid phosphatase assay kit (Sigma Chemical Co., St. Louis, MO, USA), according to the manufacturer’s instructions.

### 2.7. Animals and Diet

All animal experiments were approved by the Institutional Animal Care and Use Committee of the Konkuk University (IACUC approval number, KU 15133). Four-week-old Wistar rats were purchased from Doo Yeol Biotech (Seoul, Korea). The animals were housed in a room maintained at 22 °C, under 12-h light-dark cycles. After a one-week acclimatization period, the rats were split into 2 groups, either a sham-operated group (normal, one group, n = 12) or an OVX group (four groups, total n = 48). Ovariectomy was performed via ligation and excision of the ovaries, whereas the sham surgery involved exposing the ovaries without excision. After a one-week period of adaptation, the initial mean rat body weight was 117 ± 7 g. The sham and positive control groups were fed a normal diet (TD.97191), whereas the negative control group was fed an OVX diet (TD.95027) containing a lower calcium (0.01%) than the normal diet (0.6% of calcium) (Table 1). OVX rats fed GD collagen also received an OVX diet (TD.95027) containing a lower calcium (0.01%) content, and the added GD collagen quantity was deducted from the casein content in the OVX diet. The animals were randomly assigned to five groups (12 rats per group) as follows—(1) Sham (normal diet); (2) OVX diet (OVX + low calcium); (3) positive control (OVX + normal diet); (4) GDC2 (OVX + casein-deducted normal diet + 2 g/Kg GD collagen); and (5) GDC3 (OVX + casein-deducted normal diet + 3 g/Kg GD collagen). GD collagen was orally administered (gavage). Food intake was monitored daily, and the rats were weighed once a week. At the end of the 8-week feeding period, the rats received a lethal intraperitoneal ether overdose. Tibial bones were dissected and stored at –20 °C. Blood samples were collected from the heart under light anesthesia, and serum was then obtained via centrifugation (848× *g* for 30 min) and stored at −80 °C, prior to the biochemical assays.

### 2.8. Determination of Biochemical Variables and Estrogen Level in Serum

Serum lipid, including total cholesterol (TC), high-density lipoprotein (HDL) and triglyceride (TG) contents were measured using the T-CHO ELISA kit (3I2020; Asan Pharmaceutical, Hwaseong-si, Korea) for TC, HDL-CHO ELISA kit (3I2030; Asan Pharmaceutical, Hwaseong-si, Korea) for HDL, and the TG-S ELISA kit (3I1570; Asan Pharmaceutical, Hwaseong-si, Korea) for TG. Serum aspartate aminotransferase (AST) and alanine aminotransferase (ALT) levels were determined using an automatic biochemistry analysis system (Hitachi 7060; Hitachi, Japan). The estrogen levels in the rat serum were measured using a rat estradiol ELISA kit (Biovision Inc., Milpitas, CA, USA), according to the manufacturer’s protocol.

### 2.9. Calcium Content Measurement

Calcium content in the rat tibia was quantified using a microwave digestion system (Multiwave 3000; Anton Paar, Graz, Austria) and inductively coupled plasma mass spectrometry (HP-4500; Hewlett-Packard, Avondale, PA, USA). All experiments were performed according to official methods, using the Association of Analytical Chemists (AOAC) procedures.

### 2.10. Bone Strength Test

Tibial firmness was measured using an A/WEG wedge fracture probe (Stable Micro Systems, Godalming, UK). The tibial bone was fractured with the downward motion (3 mm/s) of a steel blade (30 mm wide). The maximum force (N) applied to break the bone was used to quantify bone firmness.

### 2.11. Micro-CT

Tibial morphometric parameters were determined in the distal tibia using a high-resolution, cone-beam micro-CT system (Inveon PET; Siemens Medical Solutions, Knoxville, TN, USA). The trabecular and cortical BMD and bone structure parameters, such as bone surface area/bone volume (BSA/BV), bone volume/total volume (BV/TV), trabecular thickness (Tb.Th), trabecular separation (Tb.Sp), and trabecular number (Tb.N), were determined by measuring trabecular bone mass and its distribution. Other parameters, such as cortical thickness (Ct.Th), were three-dimensionally determined via the measurement of cortical bone mass and its distribution, according to the standard procedures. Scans were performed using an applied voltage (80 kV) with a 1 mm aluminum filter. The image resolution of all cross-sections contained 2048 × 2048 pixels, with an isotropic voxel size of 9.31 µm. Data analysis was performed using the Inveon acquisition workplace software (Siemens). Z-scores were calculated using the following formula: Z-score = [measured BMD – age-matched BMD]/age-matched population SD [24].

### 2.12. RT-PCR Assay

Total RNA from the MG-63 cells and RAW264.7 cells that had were differentiated after 96 h, as well as tibial RNA, were isolated using TRIzol reagent (Thermo Fisher Scientific, Inc., Waltham, MA, USA). A 1-μg aliquot of total RNA was reverse-transcribed using SuperScript III Reverse Transcriptase (Invitrogen). The resultant cDNA was used for the determination of BMP-2, RUNX2, Wnt3a, osteocalcin, COL-1, OPG, RANKL, TRAP, and cathepsin K mRNA levels in the tibia, through PCR amplification, using Taq DNA polymerase (KAPA Biosystems, London, UK). As an internal control, glyceraldehyde-3-phosphate dehydrogenase (GAPDH) was used. Different primer sequences were presented as follows: GAPDH: F (5′-AACTC CCATTCCACCTT-3′), R (5′-GAGGGCCTCTCTCTTGCTCT-3′); BMP-2: F (5′-AAGGCACCCTTTGTATGTGGACT-3′), R (5′-CATGCCTTAGGGATTTTGGA-3′); RUNX2: F (5′-TCCAGCCACCTTCACTTACAC-3′), R (5′-GCGTCAACACCATCATTCTG-3′); Wnt3a: F (5′-TCCGACTCTTGGCAGAACTT-3′), R (5′-AATGGAATAGGTCCCGAACA-3′); osteocalcin: F (5′-AGCTCAACCCCAATTGTGAC-3′), R (5′-AGCTGTGCCGTCCATACTTT-3′); COL-1: F (5′-TTGACCCTAACCAAGGATGC-3′), R (5′-CACCCCTTCTGCGTTGTATT-3′); RANKL: F (5′-ACGCAGATTTGCAGGACTCGAC-3′), R (5′-TTCGTGCTCCCTCCTTTCATC-3′); TRAP: F (5′-CGCCAGAACCGTGCAGA-3′), R (5′-TCAGGCTGCTGGCTGAC-3′). The PCR products were detected by 1.2% agarose/ethidium bromide gel electrophoresis and photographed.

### 2.13. Western Blotting Analysis

Differentiated cells and tibia were homogenized in a lysis buffer containing a protease inhibitor (Roche, Mannheim, Germany) and centrifuged at refrigerated temperature (10,000 × g for 10 min at). Total protein content in the samples was quantified using a Bio-Rad DC protein assay kit. Afterward, the proteins were separated in an SDS-PAGE experiment using a 12% polyacrylamide gel. The separated proteins were transferred onto immobilon-P transfer membranes, which were blocked with 5% bovine serum albumin before incubation with specific primary antibodies against osteocalcin, COL-1, BMP-2, or RUNX2 (Abcam), SMAD5 (Santa Cruz, Texas, USA), phosphorylated serine/threonine kinase (p-AKT), p-ERK, p-p38, p-JNK, or β-actin (Cell Signaling Technology, MA, USA). The membranes were incubated with the appropriate secondary antibody, either a goat anti-rabbit IgG (H + L)-HRP conjugate or goat anti-mouse IgG (H + L)-HRP conjugate (Zymax). The immune complexes antigen–antibody were visualized using enhanced chemiluminescence. Finally, densitometric analysis of the signals was performed using a C-DiGit Blot Scanner (Li-COR, NE, USA).

### 2.14. Histology, Immunohistochemical Analysis, and TRAP Staining of Tibia

Cleaned tibias were fixed for 2 days at 40 °C in 10% neutral-buffered formalin. Decalcification was performed by immersion-stirring in 10% ethylenediaminetetraacetic acid (EDTA, pH 7.4), which was replaced daily for 20 days at room temperature (25 °C). The bones were then washed with tap water for 4 h. Afterward, the tibias were paraffin-embedded, and longitudinal 4 μm sections were cut using a microtome. Sections were mounted, stained using hematoxylin and eosin, and observed using a light microscope at a magnification of 100×.

For the immunohistochemical analysis, the decalcified and paraffin-embedded tibias were deparaffinized in xylene, and rehydrated using an ethanol series, and then rinsed in PBS. A 30 min incubation in 0.3% hydrogen peroxide was performed to quench the endogenous peroxidase activity. Next, the 4 μm sections of tibia were incubated with 10% goat serum for 30 min and incubated overnight at 4 °C, with the specific primary antibodies against RUNX2, BMP-2, Wnt3a, osteocalcin, or COL-1 (Abcam, Cambridge, MA, UK). The sections were then incubated with either a biotinylated goat anti-rabbit IgG (H + L)-horseradish peroxidase (HRP) conjugate or goat anti-mouse IgG (H + L)-HRP conjugate (Zymax, San Francisco, CA, USA). The sections were then stained with 3,3′-diaminobenzidine (DAB substrate kit; Vector Lab., CA, USA) and counterstained with hematoxylin. Negative controls were incubated with normal goat IgG instead of the primary antibody. Finally, the specimens were observed, and the images were recorded using a Nikon Eclipse TS100 microscope (Nikon, Tokyo, Japan) at 200× magnification. Data were analyzed using the Optiview 3.7 image analysis software (Korea Lab Tech, Sungnam, Korea).

Additionally, TRAP and nuclear staining of the tibia were performed using the leukocyte acid phosphatase assay kit (Sigma Chemical Co., St. Louis, MO, USA), following the manufacturer’s instructions. The specimens were examined at 200× magnification, using the Nikon Eclipse TS100 microscope.

### 2.15. Statistical Analysis

Data are presented as the mean ± standard deviation of triplicate experiments. Statistical analyses were performed using the Statistical Package for the Social Sciences (SPSS) version 18.0 (SPSS Inc., Chicago, IL, USA). Comparisons between different groups were carried out using one-way analysis of variance, followed by Duncan’s multiple range post-hoc test. *p*-values < 0.05 were considered statistically significant.

## 3. Results

### 3.1. Collagen Contents of GD

**Figure 1:**
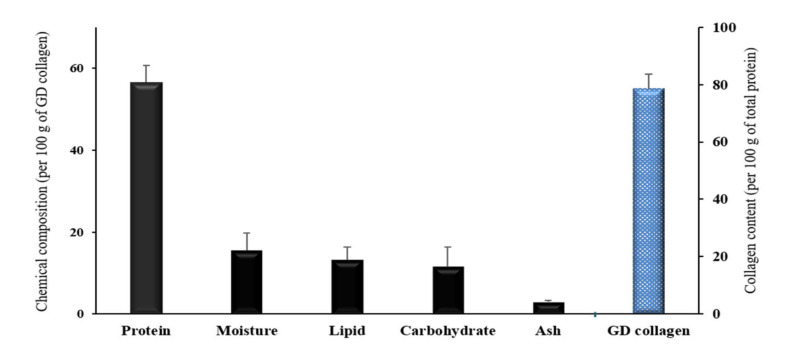
Proximate chemical composition and total collagen content of the *Gallus domesticus* (GD) collagen. Values are mean ± standard deviation of three measurements (n = 3).

The collagen contents of GD are given in Figure 1. The proximate chemical composition per 100 g of GD collagen showed 56.6% of total protein, 15.6% of moisture, 13.2% of fat, 11.6 of carbohydrate, and 2.9% of ash. Interestingly, the collagen fraction represented 78.6% of the total protein, indicating a collagen-rich product.

### 3.2. GD Collagen Regulated the Expression of Osteoblastogenesis and Mineralization in MG-63 Cells

**Figure 2:**
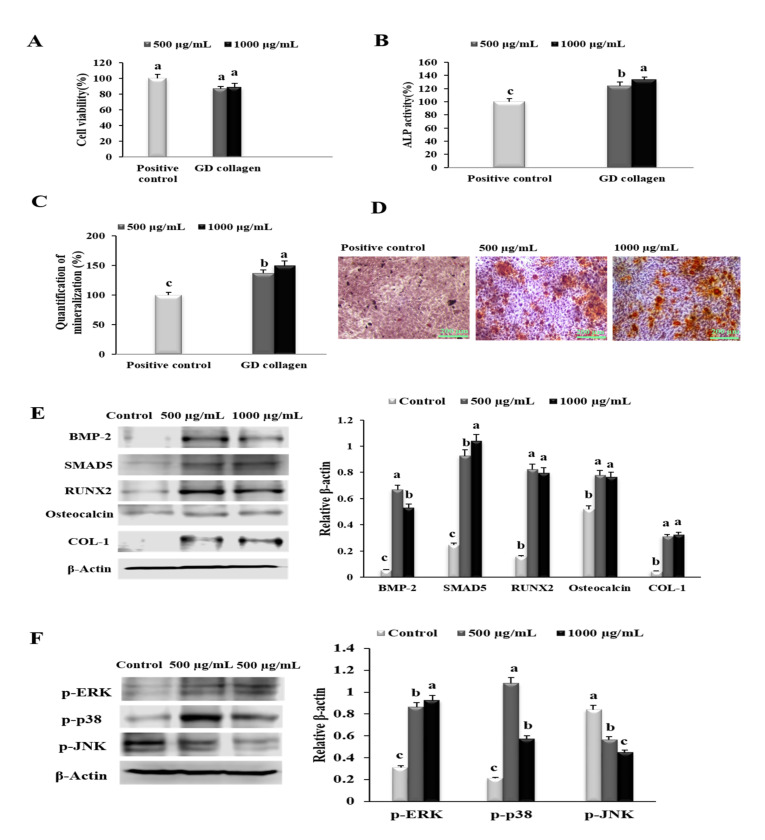
Effects of the Yeonsan Ogye chicken breed (*Gallus gallus domesticus)* collagen extract, GD collagen, on MG-63 osteoblast cell viability, alkaline phosphatase (ALP) activity, mineralization of osteoblast-secreted bone matrix, protein levels of genes involved in osteoblastic differentiation and bone nodules mineralization, and levels of p-ERK, p-p38, p-AKT, and p-JNK in MG-63 cells. (**A**) MG-63 cell viability. (**B**) ALP activity. (**C**) Quantification of mineralization. (**D**) Staining of the mineralized matrix with alizarin red S. (**E**) Protein expression of bone morphogenetic protein-2 (BMP-2), mothers against decapentaplegic homologue 5 (SMAD5), runt-related transcription factor 2 (RUNX2), osteocalcin, and type 1 collagen (COL-1) were determined by Western blot. (**F**) Levels of phosphorylated extracellular signal-regulated kinase (p-ERK), p-p38, and phosphorylated c-Jun *N*-terminal kinase (p-JNK) proteins were determined by Western blotting. Expression was quantified using ImageJ and β-actin. Values represent the mean ± standard deviation. Values with different letters were significantly different, using Duncan’s multiple range test (*p* < 0.05); (scale bar: 200 µm).

To evaluate the cytotoxicity, osteoblastogenesis and mineralization induced by GD collagen (500 and 1000 µg/mL), we investigated the effects on cell viability, ALP activity, bone matrix mineralization, and the expression of proteins in osteoblast differentiation in MG-63 cells (Figure 2). There was no significant change in MG-63 cell viability in the presence of GD collagen aqueous extracts, at all tested concentrations (Figure 2A). As shown in Figure 2B, MG-63 cells exposed to GD collagen exhibited a dose-dependent increase in ALP activity, compared to the control cells (*p* < 0.05). GD collagen significantly increased the mineralization level in the cells (Figure 2C), inducing a stronger red color in the cultured cells than in the control cells, indicating the promotion of bone mineralization (Figure 2D). Figure 2E shows that the respective concentrations (500 µg/mL and 1000 µg/mL) of GD collagen significantly increased the expression of genes involved in bone anabolism, including BMP-2 (11.9- and 9.5-fold), SMAD5 (3.7- and 4.2-fold), Runx2 (5.2- and 5.1-fold), osteocalcin (1.5- and 1.5-fold), and COL-1 (7- and 7.2-fold) in GD collagen-treated MG-63 cells, compared with the control cells (*p* < 0.05).

Next, we examined the effect of GD collagen on p-ERK, p-p38, and p-JNK expression levels using osteoblast-like MG-63 cells. As shown in Figure 2F, all tested concentrations (500 and 1000 µg/mL) significantly upregulated the levels of p-ERK (2.8- and 3.0-fold, respectively) and p-p38 (4.8- and 2.7-fold, respectively), compared with the control cells, whereas p-JNK levels were significantly inhibited (1.5- and 1.9-fold, respectively) (*p* < 0.05). Thus, GD collagen might be safe for osteoblast-like MG63 cells, as it stimulates osteoblast differentiation and bone nodule mineralization, and activates the ERK and p38 pathways.

### 3.3. GD Collagen Suppressed Osteoclast Formation and the Expression of Osteoclastogenesis-Related Genes in RAW 264.7 Cells

**Figure 3:**
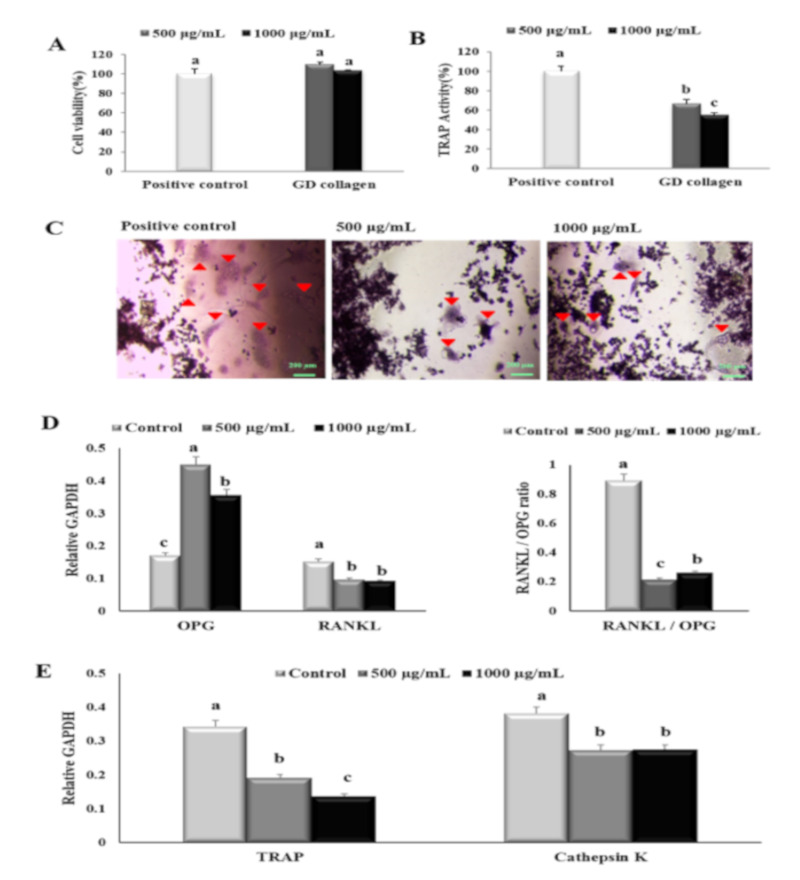
Effects of the Yeonsan Ogye chicken breed (*Gallus gallus domesticus)* collagen extract, GD collagen, on RAW 264.7 cell viability, tartrate-resistant acid phosphatase (TRAP), and expression of osteoclast differentiation specific genes. (**A**) RAW 264.7 cell viability. (**B**) TRAP activity and (**C**) staining. (**D**) mRNA expression levels of osteoprotegerin (*Opg*) and receptor activator of nuclear factor kappa-B ligand (*Rankl*), *Rankl/Opg* ratio. (**E**) mRNA expression levels of tartrate-resistant acid phosphatase (*Trap*) and cathepsin K. Expression was quantified using ImageJ, relative to that of glyceraldehyde 3-phosphate dehydrogenase (GAPDH). Values represent the mean ± standard deviation. Values with different letters were significantly different using Duncan’s multiple range test (*p* < 0.05); (scale bar: 200 µm).

To evaluate the effect of GD collagen (500 and 1000 µg/mL) on the proliferation, differentiation, and function of osteoclast precursor RAW 264.7 cells, we evaluated cell viability and identified markers for osteoclast differentiation and activity (Figure 3A). No cytotoxic effects were observed in these osteoclast precursor cells at all tested concentrations of GD collagen. Since the TRAP plays an important role in osteoclast-mediated bone resorption [25], we measured TRAP activity in RAW 264.7 cells exposed to GD collagen (Figure 3B). The results showed that GD collagen (500 and 1000 μg/mL) significantly inhibited the TRAP activity (1.5- and 1.8-fold, respectively) compared to the positive control cells (*p* < 0.05). The RAW 264.7 cells also exhibited significantly fewer TRAP-positive multinucleated cells when cultured in GD collagen-treated media, as opposed to the positive control cells (Figure 3C).

Figure 3D,E shows that the mRNA levels of the soluble decoy *Opg* receptor were significantly more upregulated in 500 μg/mL and 1000 μg/mL of GD collagen-treated cells (2.6- and 2.1-fold, respectively) than that observed in the control cells (*p* < 0.05). The mRNA levels of *Rankl* were significantly reduced to ~1.6-fold, regardless of the GD collagen dose. The *Rankl/Opg* ratio was markedly reduced at 500 µg/mL (4.2-fold) and 1000 µg/mL (3.4-fold-fold) of GD collagen, compared to the control cells (*p* < 0.05). When compared to the control cells, the GD collagen (500 μg/mL and 1000 μg/mL)-stimulated RAW 264.7 cells also exhibited a significant decrease in the mRNA levels of *Trap* (1.8- and 2.5-fold, respectively) and cathepsin K (~1.4-fold at all tested concentrations) (*p* < 0.05). These results suggested that GD collagen was less toxic to osteoclast precursors in short-time exposure, but might strongly prevent osteoclast differentiation and function.

### 3.4. GD Collagen Feed Enhanced Body Weight Gain

As shown in Table 2, all types of diets improved body weight gain in rats. However, there was no significant difference among experimental groups with regard to feed intake, body-weight gain, and food efficiency ratio (FER) (Table 2).

### 3.5. GD Collagen Regulated Serum Biochemical Parameters in OVX Rats

Lipid contents (TC, HDL, and TG), liver function parameters (AST, ALT), and estradiol levels are presented in Table 3. When compared with the sham (normal) group, TC levels significantly increased in the negative control (OVX diet group) (1.3-fold, *p* < 0.05). Interestingly, the GD collagen dosages, especially GDC2 supplementation markedly decreased the TC levels (1.3-fold), compared to the negative control group. In addition, the GDC2 effect on TC levels was slightly better than that in GDC3 and the positive control groups, and comparable to the sham (normal) group (*p* < 0.05). In the negative control group, the serum HDL levels were significantly reduced (1.4-fold), compared to the sham group (*p* < 0.05). However, the GD collagen dosages, particularly GDC2, substantially enhanced the HDL levels, compared to the negative control group (*p* < 0.05). GDC2 induced slightly better improvement in the HDL levels, compared to the GDC3 and positive control groups, and showed similar results as in the sham (normal) group. Finally, no significant difference was observed among the TG levels in the sham (normal) and the negative control groups. At the same time, the GD collagen dosages, especially the GDC3 markedly decreased the TG levels (~1.3-fold), compared to the sham (normal) and the negative control groups (*p* < 0.05).

We evaluated the effects of GD collagen treatments on the serum indices of liver function, such as AST and ALT (Table 3). There was a slight increase in the serum AST levels in the negative control group, compared to the sham (normal) group. Additionally, no significant difference was noticed, despite a minor increase in ALT. Intriguingly, the GDC2 and GDC3 diets significantly decreased the AST levels (1.3- and, 1.4-fold, respectively) compared to the negative control group (*p* < 0.05). The GDC3 inhibitory effect on AST surpassed that in the sham (normal) and the positive control groups (*p* < 0.05). Despite the small decrease in ALT levels in all GD collagen groups, compared to the negative control, no significant difference was reported.

The OVX rats showed a significant decrease in estrogen (2.5-fold), compared to the sham (normal) group (*p* < 0.05) (Table 3). However, OVX rats fed the GDC2- and GDC3-supplemented diets, showed a significant increase in estrogen levels (1.8- and 1.5-fold), respectively, compared to the negative control group (*p* < 0.05). In addition, among GD collagen dosages, GDC2 induced a greater improvement in estrogen levels, compared to the positive control (*p* < 0.05).

### 3.6. GD Collagen Increased the Calcium Content and Bone Strength of the Tibia in OVX Rats

**Figure 4:**
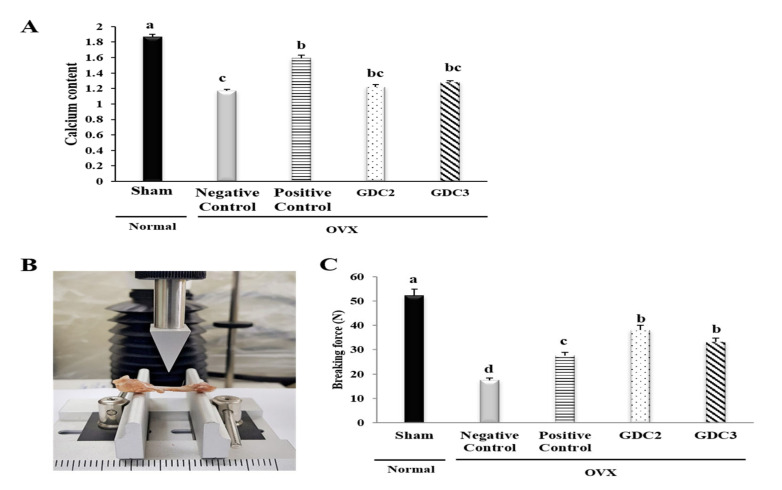
Calcium content and breaking force analysis of rat tibias. (**A**) Calcium content of rat tibias (**B**) Breaking force measurement via a texture analysis. (**C**) Breaking forces required for rat tibias. GDC2, Yeonsan Ogye flesh collagen extract (GD collagen) 2 g/Kg; GDC3, Yeonsan Ogye flesh collagen extract (GD collagen) 3 g/Kg. Values represent the mean ± standard deviation. Values with different letters differed significantly using Duncan’s multiple range test (*p* < 0.05).

Rat tibial calcium content was measured to determine the influence of GD on bone mineralization (Figure 4A). Results showed that the negative control group had a significantly lower calcium content (1.6-fold), compared with the normal (sham) group (*p* < 0.05). Interestingly, the GDC2 and GDC3 groups exhibited negligible higher calcium levels (approximately 1.04- and 1.1-fold, respectively) than the negative control group (*p* < 0.05).

No significant difference was observed among the GD collagen groups. Despite slightly lower calcium levels in the tibia of the GD collagen-fed rats, there was no significant difference compared to the positive control group.

We further investigated the effects of the GD collagen diet on bone strength. Breaking force measurements were performed using a texture analyzer (Figure 4B). We found that the force required to break the tibia was significantly lower in the negative control group (299% lower) than in the sham (normal) group. However, the breaking forces required in the GDC2 and GDC3 groups was 218% and 190% higher than in the negative control group, respectively (Figure 4C). Intriguingly, GDC2 and GDC3 exhibited significantly higher strength than the positive control group (*p* < 0.05). There was no significant difference between the GDC2 and GDC3 breaking forces. Thus, the GD collagen diet increased tibial bone strength compared to the negative control group.

### 3.7. GD Collagen Improved Bone Microarchitecture

**Figure 5:**
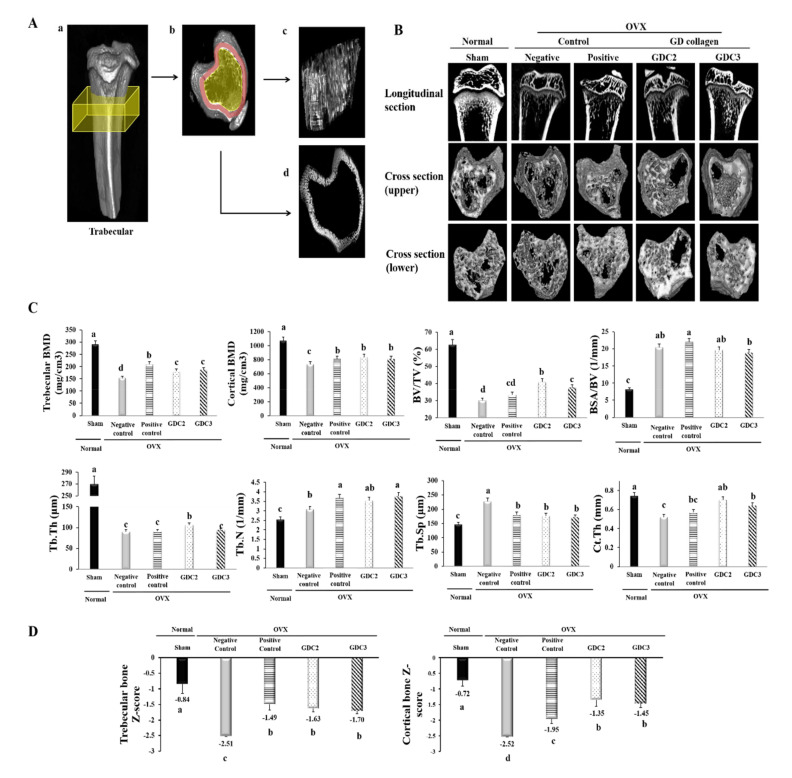
Tibial bone microstructure analysis in OVX rats. (**A**) Surface renderings show the regions used for the ex vivo analysis (yellow boxes) of the trabecular bone. (a) Shows the trabecular cross-section of the slab. (c) Shows representative isosurfaces taken from the yellow region indicated in (b). (d) Shows the surface of the cortical bone from a representative rat, which was used for cortical analysis within the red region indicated in (b). (**B**) Longitudinal section and cross-section of the trabecular bone. (**C**) Trabecular and cortical bone analysis via bone mineral density and structural parameters. (**D**) Trabecular and cortical bone Z-score measurements in OVX rats. GDC2, hydrolyzed collagen derived from GD 2 g/kg; GDC3, hydrolyzed collagen derived from GD 3 g/kg. Values represent the mean ± standard deviation. Values with different letters differed significantly using Duncan’s multiple range test (*p* < 0.05).

To evaluate the bone structure improvements induced by GD collagen diets, we performed ex vivo micro-computed tomography (micro-CT) on all animals after 8 weeks, and investigated tibial architecture using the maximum intensity projection images (Figure 5). Figure 5A illustrates the process and measurements used to analyze trabecular and cortical bones. After 8 weeks, the OVX diet rats (negative control group) showed an overall loss of tibial bone, with increased trabecular spacing, reduced bone volume, reduced trabecular number, and decreased trabecular thickness, compared to the sham group. Longitudinal sections of tibias from the negative control group showed the central spaces in the trabecular bone (Figure 5B). The upper and lower cross-sections of the tibia of the negative control group presented large spaces within the marrow, as opposed to the sham group. In contrast, longitudinal and cross-sectional analyses showed the bone marrow space was more occupied in the GDC2 and GDC3 groups than that of the negative control group. The bone structures of the GDC2 and GDC3 groups were distributed in a relatively uniform fashion to form a well-connected network, which was greater than that observed in the negative control group, and quite similar to that in the positive control (Figure 5B).

Furthermore, micro-CT analysis of the tibia metaphysis was used to determine trabecular and cortical BMD and bone structural parameters (BSA/BV, BV/TV, Tb.Th, Tb.Sp, Tb.N, and Ct.Th) (Figure 5C). Following OVX surgery, the rats exhibited significantly reduced trabecular and cortical BMD values, and BV/TV, Tb.Th, and Ct.Th, and increased BSA/BV, Tb.N, and Tb.Sp, compared to the sham group (*p* < 0.05).

Intriguingly, our data showed that the GDC2 and GDC3 rats presented higher trabecular and cortical BMD values and increased BV/TV, Tb.Th, Tb.N, and Ct.Th, compared to the negative control group. Other parameters, such as BSA/TV and Tb.Sp, were significantly lower in the GDC2 and GDC3 groups than in the negative control group (*p* < 0.05). Although there was no significant difference between the GDC2 and GDC3 overall structural parameters, GDC2 induced better BV/TV and Tb.Th than did GDC3 (*p* < 0.05). Finally, GDC2 also exhibited better bone structural change results than did the positive control group, while the GDC3 results were quite similar to those of the positive control group. These data indicated that dietary supplementation with GD collagen ameliorated the loss of trabecular bone integrity observed in OVX rats.

**Figure 6:**
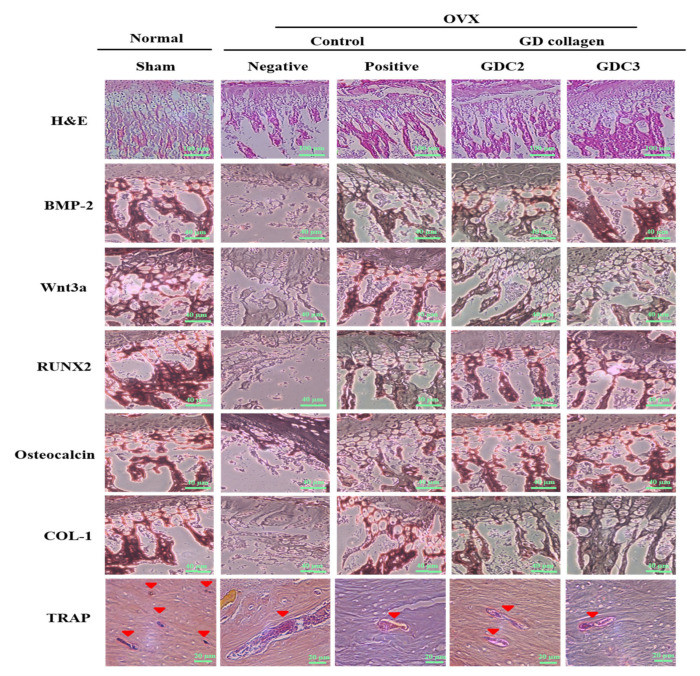
Hematoxylin and eosin (H&E) (scale bar: 100 µm), and immunohistochemical staining image of bone morphogenetic protein-2 (BMP-2), Wnt3a, runt-related transcription factor 2 (RUNX2), osteocalcin, type 1 collagen (COL-1) (scale bar: 40 µm), and tartrate-resistant acid phosphatase (TRAP) (scale bar: 20 µm). GDC2, GD collagen 2 g/Kg and GDC3, GD collagen 3 g/Kg.

Finally, we calculated the Z-score of trabecular and cortical bones (Figure 5D) in which the BMD was compared to age-matched rats. Results showed significant decreases in both trabecular and cortical bones of the negative control group, compared to the sham group (normal) (*p* < 0.05). In contrast, the OVX rats fed with GDC2 and GDC3 showed significantly increased Z-scores compared to the negative control group (*p* < 0.05), whereas no significant difference was observed among GDC2 and GDC3. In addition, no significant difference was observed between the GD collagen and the positive control group for the trabecular Z-score; whereas all GD collagen dosages displayed the highest cortical Z-score, compared to the positive control (*p* < 0.05). These findings were confirmed by a histological examination using hematoxylin and eosin staining (Figure 6).

### 3.8. GD Collagen Increased Trabecular Connections

The rat tibias were stained with hematoxylin and eosin for histopathological examination (Figure 6). As opposed to the sham group, the trabeculae of rats in the negative control group were disorderly arranged and were thinner, or completely disappeared; the remaining connections were incomplete. A non-optimal discontinuity in the trabeculae and signs of matrix resorption were apparent. In contrast, the GDC2 and GDC3 groups showed a greater number of well-connected trabeculae, arranged in a more orderly fashion than those observed in the negative control group. These findings indicated that the GD collagen diet improved the trabecular structure and continuity.

In addition, immunohistochemical staining was performed to measure the immunoreactivity of the osteogenesis-related proteins (BMP-2, Wnt3a, RUNX2, osteocalcin, and COL-1), as well as a specific marker for osteoclast function (TRAP) (Figure 6). For all osteogenesis-related proteins, the negative control group displayed mild to moderate staining, compared to the sham group. The staining for the GDC2, GDC3, and positive control groups was slightly more intense, compared to that of the negative control group. Multinucleated TRAP-positive osteoclasts were found at 8 weeks after surgery in the subchondral bone marrow space of the tibia (Figure 6). In contrast, in the negative control group, TRAP-positive osteoclasts were mainly detected in the tibial subchondral bone marrow space, as compared to the other groups. These results showed that the GD collagen diet increased the bone levels of osteogenesis-related proteins, BMP-2, RUNX2, Wnt3a, osteocalcin, and COL-1, and suppressed the mRNA expression of genes involved in osteoclastogenesis in vivo.

### 3.9. GD Collagen Promoted the Expression of Osteoblastogenesis-Related Genes

The results of the tibial mRNA expression analysis are shown in Table 4. When compared with the sham group, the mRNA levels of the negative control rats were significantly reduced for *Bmp-2* (1.8-fold), *Wnt3a* (3.3-fold), *Runx2* (6-fold), osteocalcin (1.6-fold), and *Col-1* (4-fold) (*p* < 0.05). Interestingly, compared with the negative control groups, the GDC2 and GDC3 rats showed a significant increase in *Bmp-2* (1.7- and 2.6-fold, respectively), *Wnt3a* (1.2- and 1.3-fold, respectively), *Runx2* (2.3- and 3.7-fold, respectively), osteocalcin (1.7- and 1.8-fold, respectively), and *Col-1* (2.2- and 1.3-fold, respectively). GDC3 highly promoted *Bmp-2*, *Runx2*, and osteocalcin compared with GDC2, while GDC2 greatly activated the *Col-1* gene. Overall, GD collagen dosages were more efficient in promoting *Bmp-2*, *Runx2*, and osteocalcin than was the normal diet, positive control group. These results suggested that the GD collagen diet increased the mRNA expression of osteoblastogenesis-related genes in vivo.

### 3.10. GD Collagen Inhibited Osteoclastogenesis Associated Genes

As shown in Table 4, the negative control group expressed a significantly lower *Opg* (3.7-fold), and increased *Rankl* (5.3-fold), *Rankl/Opg* ratio (20-fold), *Trap* (4.0-fold), and cathepsin K (9.5-fold), compared to the sham group (*p* < 0.05). However, compared to the negative control group, the GDC2 and GDC3 groups showed markedly enhanced OPG (3.3- and 2.6-fold, respectively) and significantly lowered levels of *Rankl* (1.6-and and 2.8-fold, respectively), *Rankl/Opg* ratio (5.4- and 7.3, respectively), *Trap* (1.7-and 1.5-fold, respectively), and cathepsin K (7.5-and 15-fold, respectively) (*p* < 0.05). Among the GD collagen dosages, GDC2 induced a higher increase in *Opg* and a better decrease in *Trap*, while GDC3 was more effective at alleviating the *Rankl/Opg* ratio, *Rankl,* and cathepsin K. The results from all GD collagen dosages were more efficient than those of the positive control group. These findings suggested that GD collagen might drive the *Rankl/Opg* ratio to inhibit the mRNA expression of osteoclastogenesis-associated genes in vivo.

### 3.11. GD Collagen Regulated MAPK Signaling

**Figure 7:**
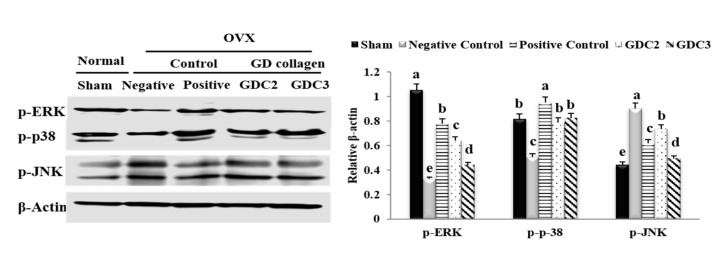
Effect of GD collagen on the MAPK signaling pathway. p-ERK, p-p38, and p-JNK protein expression in rats’ tibias were determined by Western blot analysis. GDC2, GD collagen 2 g/kg and GDC3, GD collagen 3 g/kg. Expression was quantified using ImageJ, relative to that of β-actin. The values represent the mean ± standard deviation. Values with different letters differed significantly using Duncan’s multiple range test (*p* < 0.05).

To examine the effects of GDC2 and GDC3 diets on MAPK signaling, we evaluated the activation of phosphorylated ERK, p38, and JNK in the tibia. As shown in Figure 7, the negative control group significantly inhibited p-ERK (3.2-fold) and p-p38 (1.6-fold), but promoted p-JNK (2.0-fold), compared to the sham group (*p* < 0.05). GDC2 and GDC3 significantly activated levels of p-ERK (1.9- and 1.3-fold, respectively) and p-p38 (1.5- and 1.6-fold, respectively), while deactivating p-JNK levels (1.2- and 1.8-fold, respectively) compared to the negative control group (*p* < 0.05). Both GD collagen dosages induced similar p-p38 level activation, but GDC2 induced higher p-ERK activation. GDC3 was more effective in inhibiting p-JNK. Overall, the positive control group showed higher p-ERK and p-p38 activation, compared to both GD collagen dosages; however, GDC3 was the most effective in p-ERK inhibition. Thus, the GD collagen diet modulated the MAPK signaling pathways, leading to osteoblast differentiation and inhibition of osteoclast differentiation.

## 4. Discussion

Yeonsan Ogye is a traditional Korean chicken breed, *Gallus gallus domesticus*, with the dominant gene responsible for dermal hyperpigmentation (or fibromelanosis) [26] Abnormal accumulation of eumelanin (photoprotective and antioxidant pigment) in tissues is responsible for the hyperpigmentation of Yeonsan Ogye, causing its unique dark, gray-black coloration, including black fluffy head feathers, earlobes, and pupils [27,28]. This was the first study to simultaneously examine the anti-osteoclastogenic and osteogenic effects of GD collagen (collagen extract obtained from Yeonsan Ogye flesh), via the impairment of the RANKL/OPG ratio, activation of p38 and inhibition of the JNK signaling pathways, in an OVX rat model.

Estrogen plays a key role in bone metabolism regulation by inhibiting osteoclast-mediated bone resorption and upregulating apoptosis in osteoclasts [29]. The estrogen deficiency caused by bilateral ovariectomy in rats dramatically reduced bone mineral content, density, and biomechanical strength [30,31]. The combination of ovariectomy with a low-calcium diet in rats was used to establish a rapid animal model of osteoporosis [32]. We measured the serum level of estradiol, which is the main product resulting from the biosynthesis of estrogen. Moreover, estradiol is the most potent estrogen during the premenopause period [33]. Our results showed that the additive effects of OVX and a low-calcium diet (0.01%) significantly decreased the serum estrogen levels in the negative control group. In contrast, the GD collagen supplemented the significantly enhanced estrogen levels, and these results were better than that of the OVX rats receiving a normal diet (high calcium content, 0.6%). Moreover, our results showed a significant decrease in tibial calcium levels in the negative control group, while the GD collagen diet alleviated calcium loss in rat tibia (Figure 4A). A low calcium feed might induce a decrease in calcium content in rat tibia. Kim et al. reported that when the calcium level falls due to low calcium intake and increased excretion in urine, parathyroid hormone release rises, provoking bone resorption stronger than building-up [34]. Mizuno and Kuboki showed via an in vitro study that a type I collagen supplement enhances calcium precipitation in the medium of bone marrow stromal fibroblastic cultured cells [35]. Furthermore, we demonstrated that the break force of tibias from the GDC2 and GDC3 groups was 218% and 190% higher than that required for the negative control group, respectively (Figure 4C). Our results revealed that feeding GD collagen diets to OVX rats prevented hypocalcemia and increased bone strength. Therefore, the GD collagen diet might be useful for the maintenance of bone health.

Using Micro-CT, which offers nondestructive, high-resolution 3D imaging of the internal structure of objects [36], we found that both the upper and lower cross-sections of the tibias from the negative control group exhibited fewer contents of trabecular bone, higher void spaces in the marrow area, and a rod-like structure in the bone marrow (Figure 5B). In contrast, the cross-sections of the sham (normal), the GDC2, and the GDC3 groups displayed relatively uniform patterns, well-connected networks, and better spatial distributions of a plate-like trabecular bone structure in the bone marrow. Compared with the negative control (Figure 5C), the GD collagen diets increased trabecular and cortical BMD scores, enhanced bone structure parameters (BV/TV, Tb.Th, Tb.N, and Ct.Th), and decreased other BSA/BV and Tb.Sp parameters. Additionally, trabecular and cortical bone Z-scores significantly decreased below −2.5, whereas GDC2 and GDC3 significantly increased these parameters above −1.5. In particular, all GD collagen dosages resulted in better improvement in cortical bone Z-score than the positive control. A study reported that lower Z-scores were associated with a severe osteoporotic status [37] Furthermore, a lower Z-score associated with low BMD was indicative of factors other than natural menopause and aging, which were known to adversely affect the health of the skeletal system [38]. These findings revealed an improvement in the overall bone strength and tibial trabecular bone density. These results go beyond those of previous studies [39], showing a decrease in BV/TV, Tb.Th, Tb.N, and Ct.Th, and an increase in BSA/BV and Tb.Sp, succeeding the OVX operation [39,40]. Collagen supplementation was reported to enhance BMD and increase bone strength and the external diameter of the cortical areas in the thighbone of OVX mice [17,18]. Accordingly, the improvements in bone mass and the microarchitectural network induced by the GD-collagen diet, might explain the increased biochemical strength of the bones.

**Figure 8:**
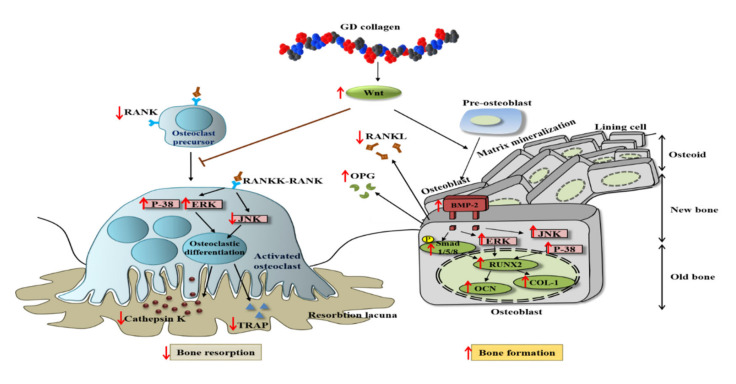
Scheme of GD collagen effects on osteoblastogenesis and osteoclastogenesis signaling pathways.

Our in vitro results demonstrated that the osteoblast precursor MG-63 cells treated with GD collagen exhibited increased ALP activity and deeply positive staining in the calcified nodules, as compared to the control cells (Figure 2B–D). Mechanistic studies revealed that the GD-collagen diet significantly upregulated the expression of osteocalcin, COL-I, Runx2, Wnt3a, BMP-2, and SMAD5, as opposed to the control cell (in vitro) and the negative control group (in vivo) (Figure 2E, Figure 6) (Table 4). Previous studies reported that enzymatically hydrolyzed collagen, gelatin, and collagen derived from tilapia, bovine, and porcine, improved various osteoblastic differentiation markers (BMP-2, Runx2, SMAD5, osteocalcin, and COL-1), increased ALP activity, and accelerated bone matrix mineralization [17,18,41,42,43]. Our findings confirmed that GDC2- and GDC3-collagen supplements promote bone formation by stimulating bone-specific matrix protein expression, bone nodule mineralization, and Wnt3a/BMP-2/SMAD5/RUNX2 anabolic signaling pathways (Figure 8).

The present study showed that the GD-collagen diet activated Wnt3a signaling and increased the synthesis of OPG in OVX rats, while decreasing the levels of RANKL, TRAP, and cathepsin K, when compared to the negative control group (Figure 8). The impairment that occurred in the RANKL/OPG ratio suggests that GD collagen supports bone formation and reduces bone resorption by inhibiting the osteoclastic differentiation [44]. Upregulation of Wnt3a expression might activate the β-catenin signals, inducing OPG synthesis by osteoblasts and bone marrow stromal cells, which in turn inhibit osteoclastic differentiation [45,46]. Synthesized soluble OPG inhibits the binding of RANKL to its RANK receptor in osteoclast precursors, thus, downregulating osteoclastic differentiation [47,48,49]. In contrast, OPG deficiency promotes RANKL-RANK binding, activating signaling pathways, and enhancing the expression of osteoclast-specific genes, such as TRAP and cathepsin K, in osteoclasts [50]. Our results showed that GDC2 and GDC3 suppressed the expression of cathepsin K, which is upregulated by RANKL and degrades bone type I collagen. These findings agree with those of Elango et al., showing that collagen treatment decreases the number of bone marrow macrophage-derived TRAP-positive osteoclasts and suppresses the expression of osteoclastogenic regulatory genes [12]. Our results suggest that GDC2 and GDC3 inhibit osteoclast formation by activating the canonical Wnt pathway, promoting OPG synthesis, and blocking the expression of RANKL, TRAP, and cathepsin K.

Studies have shown that the p38 and JNK signaling pathways are involved in the regulation of several cellular physiological functions, including cell proliferation, osteoblast differentiation, and skeletal development [8,51,52,53]. The present study found that the GD-collagen diet upregulated osteoblast-specific genes and osteoblast differentiation by activating p-38 signaling in MG-63 osteoblast cells and OVX rats (Figure 2F and Figure 7). The p38 signaling pathway is involved in osteogenesis [52], and p38 is probably involved in the regulation of COL-1 and osteocalcin expression in differentiating cells [54]. A study on the ugonin K flavonoid obtained from the roots of *Helminthostachys zeylanica* revealed that the p38 signaling pathway helps promote osteoblastic differentiation and mineralization [55]. However, OVX rats fed with GDC2 and GDC3, exhibited significantly decreased JNK expression, compared to the negative control group (Figure 7). Similarly, proanthocyanidins used as a therapeutic in an in vitro osteoporosis treatment model inhibited osteoclast formation and function by blocking the JNK signaling pathway in bone marrow macrophages and RAW264.7 cells [56]. Another report demonstrated that SP600125 suppressed PMMA particle-induced osteolysis by inhibiting the JNK signaling pathway in human osteoclast precursor cells and mouse calvaria model [57]. Thus, dietary supplementation with GD collagen might simultaneously promote osteoblast differentiation by activating the p38 signaling pathway and suppressing osteoclastogenesis by blocking the JNK signaling (Figure 8).

## 5. Conclusions

This was the first study to show that collagen extract derived from the flesh of the Yeonsan Ogye chicken breed (*Gallus domesticus*) simultaneously promoted osteoblastogenesis and prevented osteoclastogenesis both in vitro and in vivo. These findings revealed the biosafety of GD collagen. The orally administered GD collagen alleviated the severe bone calcium loss induced by a low-calcium diet intake. GD collagen enhanced BMD and bone microarchitecture, as well as improved bone structure parameters in the tibia of OVX rats fed low calcium diets. GD collagen intensified the osteoblast number and blocked osteoclastic differentiation, thereby reducing the osteoclast number. We have shown both in vitro and in vivo that GD collagen improved the expression of bone-specific matrix proteins (ALP, osteocalcin, and COL-1), Runx2 transcription factors, and osteoinductive BMP-2, and SMAD5; increased the expression of OPG; and decreased RANKL. GD collagen downregulated the expression of osteoclast-specific genes, including TRAP and cathepsin K, activated the ERK and p38 signaling pathways, and blocked the JNK pathway; it exhibited osteogenic and anti-osteoclastogenic effects. The current study provided evidence of the positive effects of GD collagen, which might help prevent and treat dysfunction in the bone remodeling process, such as osteoporosis. Future research should investigate the clinical effects of GD collagen supplements in combined estrogen deficiency- and calcium deficiency-induced bone disorders.

## Figures and Tables

**Table 1:** Feed composition.

Composition		OVX			
	Normal	Control		GD Collagen	
	Sham *	Negative **	Positive *	GDC2 **	GDC3 **
Casein (g/Kg)	200.0	200.0	200.0	198.0	197.0
L-Cystine (g/Kg)	3.0	3.0	3.0	3.0	3.0
Sucrose (g/Kg)	334.288	342.188	334.288	342.188	342.188
Corn Starch (g/Kg)	313.0	320.0	313.0	320.0	320.0
Soybean Oil (g/Kg)	60.0	60.0	60.0	60.0	60.0
Cellulose (g/Kg)	40.0	40.0	40.0	40.0	40.0
Mineral Mix, (g/Kg) ^a^	13.37	13.37	13.37	13.37	13.37
Potassium Phosphate, monobasic (g/Kg)	11.43	11.43	11.43	11.43	11.43
Vitamin Mix, (g/Kg) ^b^	10.0	10.0	10.0	10.0	10.0
Calcium (%)	0.6	0.01	0.6	0.01	0.01
Collagen (g/Kg)	-	-	-	2.0	3.0

OVX = ovariectomized; * Sham Normal, Positive Control = normal diet (TD.97191); ** Negative Control = OVX diet (low calcium diet, TD.95027); GD collagen groups = GD collagen proportion deducted from casein content in OVX diet, administration by oral gavage, GDC2, GD collagen 2 g/Kg, GDC3, GD collagen 3 g/Kg; ^a^ Mineral Mix (g/Kg) - (NaCl: 193.7325, C_6_H_7_K_3_O_8_: 575.9615, K_2_SO_4_: 136.1363, MgO: 62.8322, MnCO_3_: 9.163, C_6_H_5_FeO_7_: 15.708, ZnCO_3_: 4.1888, CuCO_3_: 0.7854, KIO_3_: 0.0262, Na_2_SeO_3_·5H_2_O: 0.0262, CrK(SO_4_)_2_·12H_2_O: 1.4399); ^b^ Vitamin Mix (g/Kg) - (p-Aminobenzoic Acid: 11.0132, Vitamin C, ascorbic acid, coated (97.5%): 101.6604, Biotin: 0.0441, Vitamin B_12_ (0.1% in mannitol): 2.9736, Calcium Pantothenate: 6.6079, Choline Dihydrogen Citrate: 349.6916, Folic Acid: 0.1982, Inositol: 11.0132, Vitamin K3, menadione: 4.9559, Niacin: 9.9119, Pyridoxine HCl: 2.2026, Riboflavin: 2.2026, Thiamin (81%): 2.2026, Vitamin A Palmitate (500,000 IU/g): 3.9648, Vitamin D3, cholecalciferol (500,000 IU/g): 0.4405, Vitamin E, DL-alpha tocopheryl acetate (500 IU/g): 24.2291, Corn Starch: 466.6878).

**Table 2:** Body weight and food intake.

		OVX			
	Normal	Control		GD Collagen	
	Sham *	Negative **	Positive *	GDC2 **	GDC3 **
Feed intake (g/day)	33.50 ± 3.64 a	34.80 ± 3.84 a	33.20 ± 3.64 a	30.90 ± 4.93 a	33.30 ± 4.74 a
Initial body weight (g)	117.33 ± 8.40 a	118.75 ± 7.68 a	118.50 ± 7.0 a	117.67 ± 6.09 a	119.00 ± 10.75 a
Final body weight (g)	339.75 ± 16.34 a	324.25 ± 30.89 a	338.25 ± 32.34 a	310.33 ± 30.11 a	355.66 ± 18.03 a
Body weight gain (g/week)	32.50 ± 15.09 a	30.81 ± 13.79 a	33.14 ± 18.28 a	28.70 ± 10.81 a	35.40 ± 17.36 a
FER ^1^	0.14 ± 0.02 a	0.13 ± 0.01 a	0.14 ± 0.02 a	0.13 ± 0.02 a	0.15 ± 0.02 a

Values represent ± standard deviation. Values with different letters differed significantly using Duncan’s multiple range test (*p* < 0.05). OVX = ovariectomized; * Sham Normal, Positive Control = normal diet (TD.97191); ** Negative Control = OVX diet (low calcium diet, TD.95027); GD collagen groups = GD collagen proportion deduced from casein content in OVX diet, administration by oral gavage, GDC2, GD collagen 2 g/Kg, GDC3, GD collagen 3 g/Kg; **^1^** FER: Food efficient ratio = body weight gain (g/week)/feed intake (g/week).

**Table 3:** Serum biochemical parameters in OVX rats.

Factors		OVX			
	Normal	Control		GD Collagen	
	Sham *	Negative **	Positive *	GDC2 **	GDC3 **
TC (mg/dL)	70.91 ± 14.01 b	90.04 ± 9.61 a	75.19 ± 6.42 ab	71.21 ± 8.80 b	82.99 ± 2.40 ab
HDL (mg/dL)	61.22 ± 4.99 a	44.51 ± 5.86 b	50.68 ± 6.07 ab	56.83 ± 2.94 a	53.45 ± 3.41 ab
TG (mg/dL)	242.72 ± 17.49 a	246.79 ± 6.14 a	210.56 ± 12.51 ab	214.85 ± 15.86 ab	181.80 ± 18.85 b
AST (IU/L)	68.47 ± 7.73 ab	76.83 ± 3.86 a	64.75 ± 3.85 b	59.35 ± 5.83 bc	53.95 ± 6.48 c
ALT (IU/L)	83.89 ± 7.82 a	95.72 ± 7.05 a	86.50 ± 3.27 a	80.24 ± 11.19 a	81.79 ± 10.72 a
Estradiol (pg/mL)	95.62 ± 4.78 a	38.81 ± 3.51 d	53.84 ± 6.39 c	70.26 ± 4.04 b	59.46 ± 3.40 bc

Values represent the mean ± standard deviation. Values with different letters differed significantly using Duncan’s multiple range test (*p* < 0.05). TC—total cholesterol; HDL—high-density lipoprotein; TC—triglyceride; AST—aspartate aminotransferase; ALT—alanine aminotransferase; OVX—ovariectomized; * Sham Normal, Positive Control = normal diet (TD.97191); ** Negative Control = OVX diet (low-calcium diet, TD.95027); GD collagen groups = GD collagen proportion deduced from casein content in OVX diet, administration by oral gavage, GDC2, GD collagen 2 g/kg, GDC3, and GD collagen 3 g/kg.

**Table 4:** The Effect of GD collagen on mRNA expression levels in the tibia of OVX rats through reverse transcriptase-polymerase chain reaction (RT–PCR) analysis.

Factors (Relative GAPDH)		OVX			
	Normal	Control		GD collagen	
	Sham *	Negative **	Positive *	GDC2 **	GDC3 **
*Bmp-2*	0.202 ± 0.01 b	0.112 ± 0.01 d	0.152 ± 0.01 c	0.189 ± 0.01 b	0.295 ± 0.02 a
*Wnt3a*	0.886 ± 0.04 a	0.274 ± 0.02 d	0.507 ± 0.03 b	0.320 ± 0.02 c	0.342 ± 0.02 c
*Runx2*	0.734 ± 0.04 a	0.116 ± 0.01 d	0.224 ± 0.01 c	0.284 ± 0.02 b	0.438 ± 0.02 a
Osteocalcin	0.939 ± 0.05 b	0.567 ± 0.03 d	0.803 ± 0.05 c	0.969 ± 0.05 ab	1.053 ± 0.06 a
*Col-1*	0.772 ± 0.04 a	0.194 ± 0.01 d	0.561 ± 0.03 b	0.421 ± 0.02 c	0.249 ± 0.02 d
*Opg*	1.002 ± 0.06 a	0.266 ± 0.02 e	0.556 ± 0.04 d	0.885 ± 0.05 b	0.686 ± 0.04 c
*Rankl*	0.212 ± 0.02 e	1.113 ± 0.06 a	0.955 ± 0.06 b	0.692 ± 0.04 c	0.393 ± 0.03 d
*Rankl/Opg*	0.211 ± 0.01 e	4.186 ± 0.21 a	1.716 ± 0.09 b	0.781 ± 0.05 c	0.572 ± 0.03 d
*Trap*	0.259 ± 0.02 d	1.034 ± 0.06 a	0.736 ± 0.04 b	0.589 ± 0.03 c	0.677 ± 0.04 b
Cathepsin K	0.113 ± 0.01 d	1.050 ± 0.06 a	0.683 ± 0.04 b	0.142 ± 0.01 c	0.065 ± 0.01 e

Values represent the mean ± standard deviation. Values with different letters differed significantly using Duncan’s multiple range test (*p* < 0.05). Osteoblastogenesis-related genes: *Bmp-2*, *Wnt3a*, *Runx2*, osteocalcin, and *Col-1*. Osteoclastogenesis-related genes: *Opg*, *Rankl*, *Trap*, and cathepsin K. *Rankl/Opg* ratio was derived from mRNA expression levels. GDC2, hydrolyzed collagen derived from GD 2 g/kg; and GDC3, hydrolyzed collagen derived from GD 3 g/kg. The expression was quantified using ImageJ, relative to glyceraldehyde 3-phosphate dehydrogenase (GAPDH). OVX = ovariectomized; * Sham Normal, Positive Control = normal diet (TD.97191); ** Negative Control = OVX diet (low calcium diet, TD.95027); GD collagen groups = GD collagen proportion deduced from casein content in OVX diet, administration by oral gavage, GDC2, GD collagen 2 g/kg, GDC3, GD collagen 3 g/kg.

## References

[1] Florencio-Silva R., Sasso G.R., Sasso-Cerri E., Simões M.J., Cerri P.S. (2015). Biology of Bone Tissue: Structure, Function, and Factors That Influence Bone Cells. Biomed. Res. Int..

[2] Craig L.E., Dittmer K.E., Thompson K.G. (2015). Bones and Joints. Jubb, Kennedy & Palmer’s Pathology of Domestic Animals.

[3] Khajuria D.K., Razdan R., Mahapatra D.R. (2015). Drugs for the management of osteoporosis: A review. Rev. Bras. Reumatol..

[4] Hall G.M., Daniels M., Doyle D.V., Spector T.D. (1994). Effect of hormone replacement therapy on bone mass in rheumatoid arthritis patients treated with and without steroids. Arthritis Rheum..

[5] Maĭchuk E., Voevodina I.V., Mitrokhina T.V., Makarova I.A., Iurenev S.V. (2014). The risk of atherosclerosis and osteoporosis development in post-ovariectomy syndrome women during hormone replacement therapy. Ter. Arkh..

[6] Sieberath A., Della Bella E., Ferreira A.M., Gentile P., Eglin D., Dalgarno K. (2020). A Comparison of Osteoblast and Osteoclast In Vitro Co-Culture Models and Their Translation for Preclinical Drug Testing Applications. Int. J. Mol. Sci..

[7] Gennari L., Rotatori S., Bianciardi S., Gonnelli S., Nuti R., Merlotti D. (2015). Appropriate models for novel osteoporosis drug discovery and future perspectives. Expert Opin. Drug Discov..

[8] An J., Yang H., Zhang Q., Liu C., Zhao J., Zhang L., Chen B. (2016). Natural products for treatment of osteoporosis: The effects and mechanisms on promoting osteoblast-mediated bone formation. Life Sci..

[9] Burt L.A., Billington E.O., Rose M.S., Raymond D.A., Hanley D.A., Boyd S.K. (2019). Effect of High-Dose Vitamin D Supplementation on Volumetric Bone Density and Bone Strength: A Randomized Clinical Trial. JAMA.

[10] Choi H.K., Kim G.J., Yoo H.S., Song D.H., Chung K.H., Lee K.J., Koo Y.T., An J.H. (2019). Vitamin C Activates Osteoblastogenesis and Inhibits Osteoclastogenesis via Wnt/β-Catenin/ATF4 Signaling Pathways. Nutrients.

[11] Jin Y.R., Stohn J.P., Wang Q., Nagano K., Baron R., Bouxsein M.L., Rosen C.J., Adarichev V.A., Lindner V. (2017). Inhibition of osteoclast differentiation and collagen antibody-induced arthritis by CTHRC1. Bone.

[12] Elango J., Sanchez C., de Val J.E.M.S., Henrotin Y., Wang S., Motaung K.S.C.M., Guo R., Wang C., Robinson J., Regenstein J.M. (2018). Cross-talk between primary osteocytes and bone marrow macrophages for osteoclastogenesis upon collagen treatment. Sci. Rep..

[13] Nagai T., Suzuki N. (2000). Isolation of collagen from fish waste material-skin, bone and fins. Food Chem..

[14] Nomura Y., Oohashi K., Watanabe M., Kasugai S. (2005). Increase in bone mineral density through oral administration of shark gelatin to ovariectomized rats. Nutrition.

[15] Adam M., Spacek P., Hulejová H., Galiánová A., Blahos J. (1996). Postmenopausal osteoporosis. Treatment with calcitonin and a diet rich in collagen proteins. Cas. Lek. Ceskysh.

[16] Xu D., Shen W. (2007). Chicken collagen type II reduces articular cartilage destruction in a model of osteoarthritis in rats. West Indian Med. J..

[17] Guillerminet F., Beaupied H., Fabien-Soulé V., Tomé D., Benhamou C.L., Roux C., Blais A. (2010). Hydrolyzed collagen improves bone metabolism and biomechanical parameters in ovariectomized mice: An in vitro and in vivo study. Bone.

[18] De Almeida Jackix E., Cúneo F., Amaya-Farfan J., de Assunção J.V., Quintaes K.D. (2010). A food supplement of hydrolyzed collagen improves compositional and biodynamic characteristics of vertebrae in ovariectomized rats. J. Med. Food..

[19] Porfírio E., Fanaro G.B. (2016). Collagen supplementation as a complementary therapy for the prevention and treatment of osteoporosis and osteoarthritis: A systematic review. Rev. Bras. Geriatr. Gerontol..

[20] AOAC (2000). Official Methods of Analysis of the AOAC.

[21] Tabakaeva O.V., Tabakaev A.V., Piekoszewski W. (2018). Nutritional composition and total collagen content of two commercially important edible bivalve molluscs from the Sea of Japan coast. J. Food Sci. Technol..

[22] Ignat’eva N.Y., Danilov N.A., Averkiev S.V., Obrezkova M.V., Lunin V.V., Sobol’ E.N. (2007). Determination of hydroxyproline in tissues and the evaluation of the collagen content of the tissues. J. Anal. Chem..

[23] Yoo H.S., Chung K.H., Lee K.J., Kim D.H., An J.H. (2015). Effect of *Gallus gallus* var. *domesticus* (Yeonsan ogolgye) extracts on osteoblast differentiation and osteoclast formation. Microbiol. Biotechnol. Lett..

[24] Park K.H., Lim J.S., Kim K.M., Rhee Y., Lim S.K. (2016). Z-score discordance and contributing factors in healthy premenopausal women with low bone mineral density: The Korean National Health and Nutrition Examination Survey 2008–9. J. Bone Miner. Metab..

[25] Harada K., Itoh H., Kawazoe Y., Miyazaki S., Doi K., Kubo T., Akagawa Y., Shiba T. (2013). Polyphosphate-mediated inhibition of tartrate-resistant acid phosphatase and suppression of bone resorption of osteoclasts. PLoS ONE.

[26] Sohn J.I., Nam K., Hong H., Kim J.M., Lim D., Lee K.T., Do Y.J., Cho C.Y., Kim N., Chai H.H. (2018). Whole genome and transcriptome maps of the entirely black native Korean chicken breed *Yeonsan Ogye*. GigaScience.

[27] Lukanov H., Genchev A. (2013). Fibromelanosis in domestic chickens, Review. Agric. Sci. Technol..

[28] Liberti D., Alfieri M.L., Monti D.M., Panzella L., Napolitano A. (2020). A Melanin-Related Phenolic Polymer with Potent Photoprotective and Antioxidant Activities for Dermo-Cosmetic Applications. Antioxidants.

[29] Kameda T., Mano H., Yuasa T., Mori Y., Miyazawa K., Shiokawa M., Nakamaru Y., Hiroi E., Hiura K., Kameda A. (1997). Estrogen inhibits bone resorption by directly inducing apoptosis of the bone-resorbing osteoclasts. J. Exp. Med..

[30] Zhang L.Z., Xin J.L., Zhang X.P., Fu Q., Zhang Y., Zhou Q.L. (2013). The anti-osteoporotic effect of velvet antler polypeptides from Cervus elaphus Linnaeus in ovariectomized rats. J. Ethnopharmacol..

[31] Mori-Okamoto J., Otawara-Hamamoto Y., Yamato H., Yoshimura H. (2004). Pomegranate extract improves a depressive state and bone properties in menopausal syndrome model ovariectomized mice. J. Ethnopharmacol..

[32] Gao X., Ma W., Dong H., Yong Z., Su R. (2014). Establishing a rapid animal model of osteoporosis with ovariectomy plus low calcium diet in rats. Int. J. Clin. Exp. Pathol..

[33] Cui J., Shen Y., Li R. (2013). Estrogen synthesis and signaling pathways during aging: From periphery to brain. Trends Mol. Med..

[34] Kim C., Park D. (2013). The effect of restriction of dietary calcium on trabecular and cortical bone mineral density in the rats. J. Exerc. Nutr. Biochem..

[35] Mizuno M., Kuboki Y. (2001). Osteoblast-Related Gene Expression of Bone Marrow Cells during the Osteoblastic Differentiation Induced by Type I Collagen. J. Biochem..

[36] Liu H., Li W., Liu Y.S., Zhou Y.S. (2016). Bone micro-architectural analysis of mandible and tibia in ovariectomised rats: A quantitative structural comparison between undecalcified histological sections and micro-CT. Bone Joint Res..

[37] Govindarajan P., Schlewitz G., Schliefke N., Weisweiler D., Alt V., Thormann U., Lips K.S., Wenisch S., Langheinrich A.C., Zahner D. (2013). Implications of combined ovariectomy/multi-deficiency diet on rat bone with age-related variation in bone parameters and bone loss at multiple skeletal sites by DEXA. Med. Sci. Monit. Basic Res..

[38] Tatara M.R., Krupski W., Majer-Dziedzic B. (2017). Bone mineral density changes of lumbar spine and femur in osteoporotic patient treated with bisphosphonates and beta-hydroxy-beta-methylbutyrate (HMB): Case report. Med. Balt..

[39] Li X., Ominsky M.S., Warmington K.S., Morony S., Gong J., Cao J., Gao Y., Shalhoub V., Tipton B., Haldankar R. (2009). Sclerostin antibody treatment increases bone formation, bone mass, and bone strength in a rat model of postmenopausal osteoporosis. J. Bone Miner. Res..

[40] Peng S., Zhang G., He Y., Wang X., Leung P., Leung K., Qin L. (2009). Epimedium-derived flavonoids promote osteoblastogenesis and suppress adipogenesis in bone marrow stromal cells while exerting an anabolic effect on osteoporotic bone. Bone.

[41] Liu C., Sun J. (2015). Hydrolyzed tilapia fish collagen induces osteogenic differentiation of human periodontal ligament cells. Biomed. Mater..

[42] Liu J., Zhang B., Song S., Ma M., Si S., Wang Y., Xu B., Feng K., Wu J., Guo Y. (2014). Bovine collagen peptides compounds promote the proliferation and differentiation of MC3T3-E1 pre-osteoblasts. PLoS ONE.

[43] Zhu L., Xie Y., Wen B., Ye M., Liu Y., Imam K.M.S.U., Cai H., Zhang C., Wang F., Xin F. (2020). Porcine bone collagen peptides promote osteoblast proliferation and differentiation by activating the PI3K/Akt signaling pathway. J. Funct. Foods.

[44] Kapasa E.R., Giannoudis P.V., Jia X., Hatton P.V., Yang X.B. (2017). The Effect of RANKL/OPG Balance on Reducing Implant Complications. J. Funct. Biomater..

[45] Glass D.A., Bialek P., Ahn J.D., Starbuck M., Patel M.S., Clevers H., Taketo M.M., Long F., McMahon A.P., Lang R.A. (2005). Canonical Wnt signaling in differentiated osteoblasts controls osteoclast differentiation. Dev. Cell..

[46] Kobayashi Y., Uehara S., Koide M., Takahashi N. (2015). The regulation of osteoclast differentiation by Wnt signals. Bonekey Rep..

[47] Lacey D.L., Timms E., Tan H.L., Kelley M.J., Dunstan C.R., Burgess T., Elliott R., Colombero A., Elliott G., Scully S. (1998). Osteoprotegerin ligand is a cytokine that regulates osteoclast differentiation and activation. Cell.

[48] Fazzalari N.L., Kuliwaba J.S., Atkins G.J., Forwood M.R., Findlay D.M. (2001). The ratio of messenger RNA levels of receptor activator of nuclear factor kappaB ligand to osteoprotegerin correlates with bone remodeling indices in normal human cancellous bone but not in osteoarthritis. J. Bone Miner. Res..

[49] Min H., Morony S., Sarosi I., Dunstan C.R., Capparelli C., Scully S., Van G., Kaufman S., Kostenuik P.J., Lacey D.L. (2000). Osteoprotegerin reverses osteoporosis by inhibiting endosteal osteoclasts and prevents vascular calcification by blocking a process resembling osteoclastogenesis. J. Exp. Med..

[50] Kim J.H., Kim E.Y., Lee B., Min J.H., Song D.U., Lim J.M., Eom J.W., Yeom M., Jung H.S., Sohn Y. (2016). The effects of lycii radicis cortex on RANKL-induced osteoclast differentiation and activation in RAW 264.7 cells. Int. J. Mol. Med..

[51] Eddie R.C., Beatriz G., Francesc V. (2016). p38 MAPK signaling in osteoblast differentiation. Front. Cell Dev. Biol..

[52] Thouverey C., Caverzasio J. (2015). Focus on the p38 MAPK signaling pathway in bone development and maintenance. Bonekey Rep..

[53] Rodríguez-Carballo E., Gámez B., Sedó-Cabezón L., Sánchez-Feutrie M., Zorzano A., Manzanares-Céspedes C., Rosa J.L., Ventura F. (2014). The p38α MAPK function in osteoprecursors is required for bone formation and bone homeostasis in adult mice. PLoS ONE.

[54] Suzuki A., Guicheux J., Palmer G., Miura Y., Oiso Y., Bonjour J.P., Caverzasio J. (2002). Evidence for a role of p38 MAP kinase in expression of alkaline phosphatase during osteoblastic cell differentiation. Bone.

[55] Lee C.H., Huang Y.L., Liao J.F., Chiou W.F. (2011). Ugonin K promotes osteoblastic differentiation and mineralization by activation of p38 MAPK- and ERK-mediated expression of Runx2 and osterix. Eur. J. Pharmacol..

[56] Zhu W., Yin Z., Zhang Q., Guo S., Shen Y., Liu T., Liu B., Wan L., Li S., Chen X. (2019). Proanthocyanidins inhibit osteoclast formation and function by inhibiting the NF-κB and JNK signaling pathways during osteoporosis treatment. Biochem. Biophys. Res. Commun..

[57] Yamanaka Y., Clohisy J.C., Matsuno T., Abu-Amer Y. JNK inhibitor, SP600125 blocks PMMA Particle-Induced Osteolysis in Human osteoclast precursor cells and Mice Calvaria Model. Proceedings of the 55th Annual Meeting of the Orthopaedic Research Society.

